# Impact of percutaneous coronary intervention of chronic total occlusion on left ventricular function using cardiac magnetic resonance imaging

**DOI:** 10.1186/1532-429X-13-S1-M6

**Published:** 2011-02-02

**Authors:** Gideon A Paul, Kim Connelly, Mo Zia, Alexander J Dick, Brad H Strauss, Graham A Wright

**Affiliations:** 1 Kings College Hospital, London, UK; 2 St Michaels Hospital, Toronto, ON, Canada; 3 Sunnybrook Health Sciences Centre, Toronto, ON, Canada; 4 University of Toronto, Toronto, ON, Canada

## Objective

To assess the role of CMR in the treatment of true chronic total occlusions (CTO) with percutaneous coronary intervention (PCI) and drug eluting stent implantation.

## Introduction

Successful PCI for CTO may confer an improved prognosis and a reduction in major adverse cardiac events. However most trials have included occlusions of short duration (less than 4 weeks). In this study we assessed the impact of PCI on LV function in patients with true CTOs (TIMI flow grade 0 and greater than 12 weeks duration) using serial CMR imaging as well as the predictive value of late gadolinium enhancement when performed prior to revascularization.

## Methods

Thirty patents referred for PCI to a single vessel CTO underwent CMR examination prior to and six months after PCI. Technical success was defined as recanalization of the occluded vessel and DES implantation with a final residual diameter stenosis <30%. LV function and infarct size were assessed using a 1.5T GE MRI system. Segmental wall thickening (SWT) was measured within the perfusion territory of the CTO using the 16-segment model and segments were dysfunctional if the SWT was ≤45%. The transmural extent of infarction (TEI) was calculated by dividing the hyperenhanced area by the total area x 100; a score of ≤25% were considered viable.

## Results

**Figure 1:**
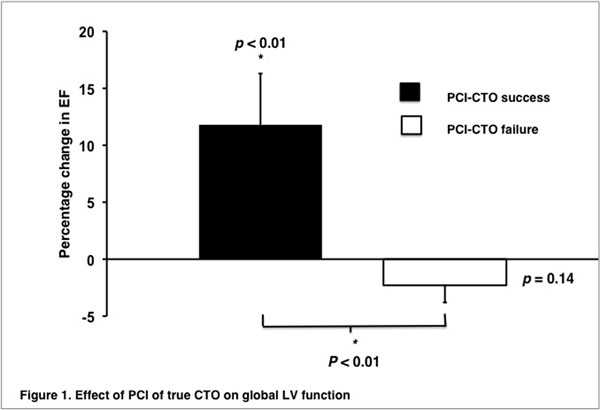
Effect of PCI of true CTO on global LV function

**Figure 2:**
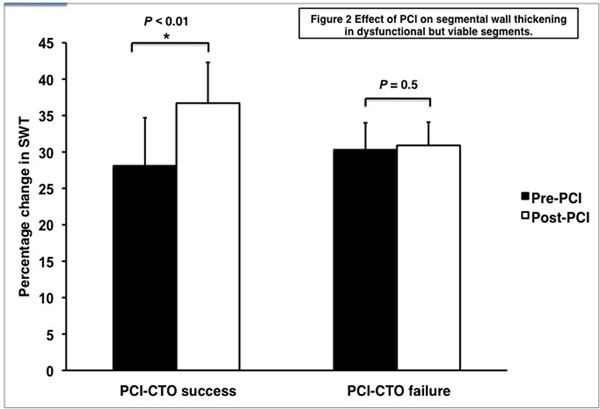
Effect of PCI on segmental wall thickening in dysfunctional but viable segments

Technical success was achieved in 19 of the 30 patients (63%). CTO duration was greater in patients with failed revascularization but other baseline demographics were well matched between groups (Table 1). PCI-CTO success resulted in a significant increase in LVEF when compared to both baseline (50 ± 13 vs 54 ± 11; P < 0.01) and with PCI-CTO failure (11.8 ± 19.8 vs -2.3 ± 5.1, p < 0.01, Figure 1). In dysfunctional but viable segments only PCI success conferred a significant improvement in SWT compared to baseline (26 ± 6 vs 40 ± 10; P < 0.001, Figure 2). There were no episodes of major adverse cardiac events in either group at 21 months follow up.

**Table 1:** Baseline demographics

	Total (n=30)	CTO-PCI success (n=19)	CTO-PCI failure (n=11)	P-value
Age/ years	62.2 ± 10.2	62.4 ± 9.8	61.8 ± 11.4	0.89

Male,n (%)	25 (83)	14 (74)	11 (100)	0.13

CCSA anginal class	2.13 ± 0.68	2.21 ± 0.63	2.0 ± 0.77	0.42

LVEF/ %	53.0 ± 11.6	50.3 ± 12.6	57.6 ± 8.1	0.09

CTO duration, months	36.9 ± 70.8	12.6 ± 26.4	78.8 ± 101.1	0.01

Vessel, n (%)				0.35
RCA	16 (53)	9 (47)	7 (64)	
LAD	11 (37)	7 (37)	4 (36)	
LCx	3 (10)	3 (16)	0	

Prior MI, n (%)	17 (59)	11 (58)	6 (56)	0.61

Diabetes mellitus, n (%)	7 (23)	5 (26)	2 (18)	0.61

Hypertension	23 (77)	14 (74)	9 (82)	0.61

## Conclusion

PCI-CTO success of true CTOs can improve global LV function. The TEI, assessed with CMR, can be used to help predict improvements in regional wall function. Failed PCI was not associated with increased MACE at medium-term follow up.

